# Mediastinal high-grade vasculogenic mesenchymal tumour with seminoma: a case report and literature review

**DOI:** 10.1186/s13000-023-01333-9

**Published:** 2023-04-28

**Authors:** Shang Zhanxian, Han Yuchen, Wei Jinzhi, Zhu Lei

**Affiliations:** Department of Pathology, Shanghai Chest Hospital, Shanghai Jiao Tong University School of Medicine, Shanghai, 200030 China

**Keywords:** Vasculogenic mesenchymal tumour, seminoma, Germ cell tumours with somatic-type solid malignancy, Morning glory syndrome.

## Abstract

Germ cell tumours with somatic-type solid malignancy (GCT-STM) are a rare disease of the mediastinum. Recently, a cohort of vasculogenic mesenchymal tumour (VMT)-nonseminoma cases with different prognoses were recognized and reported. Here, we report a case of mediastinal high-grade VMT with a seminoma. A 16-year-old male had a fever, chest tightness and fatigue. Chest CT showed a 7.5 cm×5.3 cm solid mass in the right anterior mediastinum. The serum levels of alpha-fetoprotein (AFP), beta-human chorionic gonadotropin (β-HCG) and carcinoembryonic antigen (CEA) were within the normal range. Tumorectomy was performed. The tumour was irregular, and no capsule was found. The cut surface was greyish white and greyish brown with medium consistency. There were foci of bleeding and necrosis. Microscopic histology showed prominent vascular proliferation, which was lined by mildly atypical endothelial cells in a cellular stroma with significant cytologic atypia. The vascular spectrum varied from crevice-like or antler-like thin- to thick-walled vessels. Beyond the tumour area, inside the remnant thymus tissues, there were small clusters of polygonal tumour cells with clear cytoplasm, distinct cell membranes, and round to polygonal nuclei with prominent nucleoli that were positive for Oct4, PLAP, SALL4 and CD117. The patient did not receive any treatments pre- or postoperation, and his condition was stable without progression after 14 months of follow-up evaluation. Here, we added a new entity of GCT-STM of the mediastinum composed of VMT and seminoma. A better understanding of the pathological features of GCT-VMT could help pathologists improve their awareness of these rare diseases.

## Background

Germ cell tumours with somatic-type solid malignancy (GCT-STM) of the mediastinum are mediastinal germ cell tumours with a malignant neoplasm resembling those seen at somatic sites. Somatic-type malignancy is most commonly associated with mature teratoma and less commonly associated with immature teratoma, choricarcinomas, yolk sac tumours, and embryonal carcinoma, but it is not associated with seminomas [1, 2]. Somatic-type tumours include sarcomas and carcinomas, of which rhabdomyosarcoma is the most frequent. Angiosarcoma and leiomyosarcoma have also been reported. In 2021, Levy et al. reported 55 postchemotherapy cases of vasculogenic mesenchymal tumours of the mediastinum, which had a distinctive neoplasm originating from mediastinal yolk sac tumours. They proposed the concept of vasculogenic mesenchymal tumour (VMT), which is an occasional precursor to angiosarcoma [3]. The VMT represented a developed angiogenic lineage from immature to well-developed neoplastic vessels within the primitive mesenchyme.

Here, we report a case of a mediastinal tumour that contains both high-grade VMT and seminoma without any previous treatment, and review the relevant literature to further recognize GCT-VMT.

## Case presentation

**Fig. 1:**
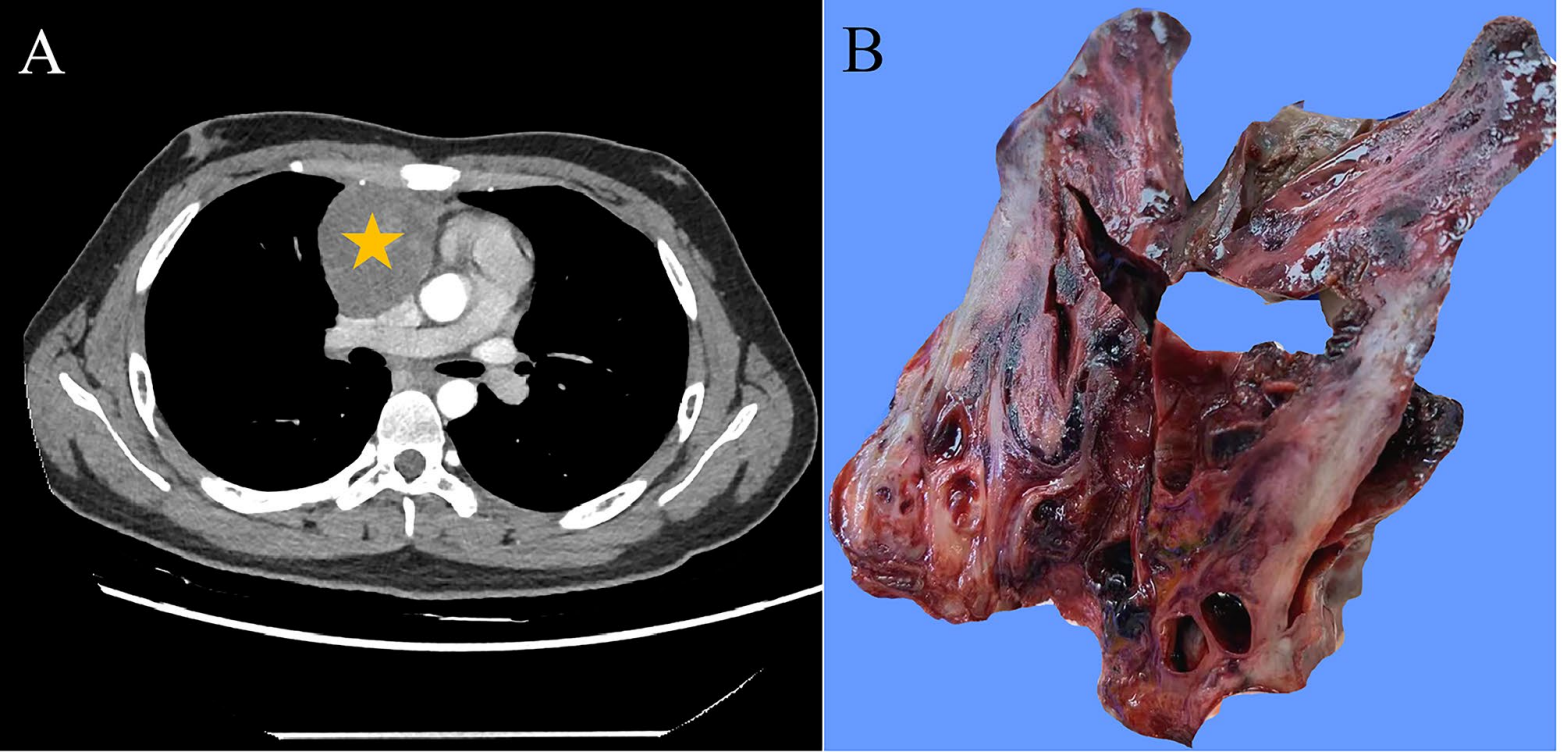
Imaging and gross examination of the mediastinal tumour. (A) CT demonstrates a solid mass (yellow star) in the right anterior mediastinum with uneven density and well-defined boundaries. (B) The cut surface of the tumour was greyish‒white solid mixed with haemorrhagic cystic changes

The 16-year-old male patient had a fever, in which the highest temperature was 38 °C, chest tightness and fatigue for one week. Chest CT showed an oval-shaped solid mass with irregular margins in the right anterior mediastinum, and the adjacent great vessels were compressed and displaced from normal sites (Fig. 1A). Serological analysis results were as follows: AFP, 1.24 ng/ml; β-HCG, < 0.6 ng/ml; CEA, 2.49 ng/ml; and lactate dehydrogenase, 256 U/L. The patient underwent tumorectomy, and no further treatment was given after the operation. The patient had been diagnosed with morning glory syndrome (MGS) in his childhood.

## Pathological findings

An irregular mediastinal mass measuring approximately 7.6 cm × 5.5 cm × 5.0 cm with no capsule was found. The cut surface was mostly greyish brown with haemorrhagic cysts and foci of necrosis, and the consistency was medium. The tumours were cut at 0.5 cm intervals and sampled completely (Fig. 1B).

**Fig. 2:**
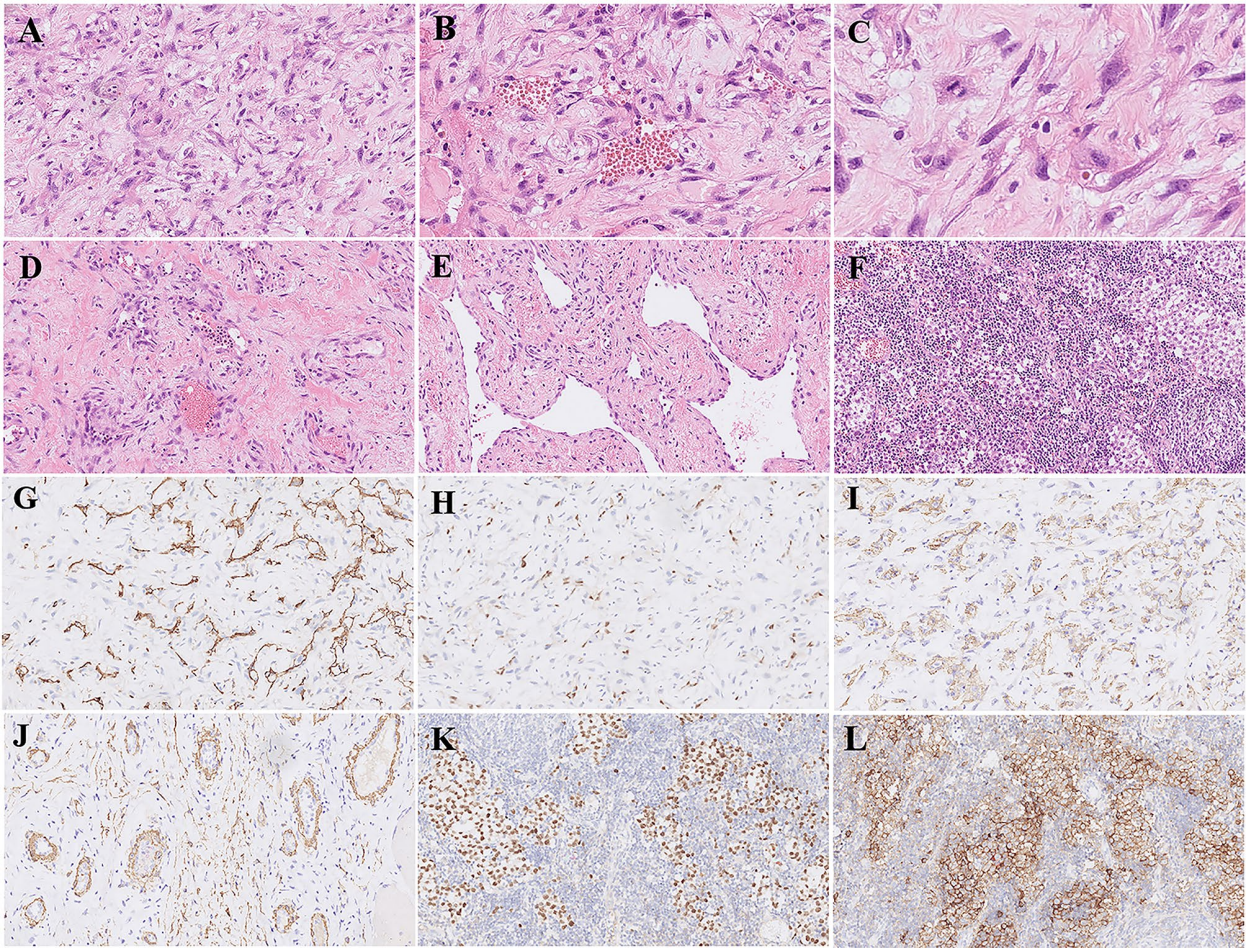
Microscopic morphology and immunohistochemical characteristics of the tumour. (A) Spindle to stellate cells in a myxoid stroma. (B) Atypical cells form cleft-like spaces in which erythrocyte clusters can be seen. (C) Mitosis and individual erythrocytes within intracytoplasmic lumens. (D) Thin-walled blood vessels were formed. (E) Plexuses of vessels were anastomosed with a collagenized stroma. (F) Atypical seminoma cell clusters with clear cytoplasm were seen in the lymphoid stroma. (G) Spindle cells lining along clefts in myxoid stroma were positive for CD34. (H) ERG was expressed in endothelial cells. (I) The stellate cells around the clefts expressed SMA, while the lining cells were negative. (J) SMA expression was observed around the thin-walled small blood vessels, whereas the endothelial cells were negative. K) SALL4 was positive in the seminoma. L) PLAP was positive in the seminoma

Histologically, most of the tumour was haemorrhagic and necrotic, and a substantial region was mainly composed of a spectrum of vascular proliferation. In addition to the reticulated vessels, the myxoid cellular foci were highly remarkable. In this area, spindle to stellate cells were in a typical myxoid stroma (Fig. 2A). In some other areas, vasculogenesis occurred, and spindled to stellate stromal cells were arranged around small aggregates of erythrocytes in vessels (Fig. 2B). All stromal cells were atypical and mitotic, and some of them formed cleft-like spaces. Infrequently, atypical stromal cells that were found contained individual erythrocytes within intracytoplasmic lumens (Fig. 2C). Around all well-defined walls of vessels, smooth muscle differentiation was defined with atypia, while the nonstratified endothelium lining the lumen of these thick-walled neoplastic vessels lacked atypia (Fig. 2D). In the wider region with a collagenous stroma, well-defined plexuses of incipient vessels anastomosed (Fig. 2E). Unlike angiosarcomas, the endothelial cells of these blood vessels were less atypical than the associated stromal cells. Although no angiosarcoma existed in this case, atypical mitoses were easily found, indicating that it was a high-grade VMT. Immunohistochemical staining made the entire spectrum of vascular proliferation more visible. The surface endothelium of clefts in the myxoid stroma was highly positive for CD34 (Fig. 2G). In the same area, focal and weaker reactivity for ERG was present (Fig. 2H). In well-differentiated regions of the vessel, CD34 and ERG were positive in the endothelium rather than stromal cells. Both cleft-like spaces (Fig. 2I) and thin-walled vessels (Fig. 2J) showed a subendothelial linear pattern of reactivity for SMA, which suggests that these cells have smooth muscle differentiation. Staining for cytokeratin AE1/AE3 was negative in all tumour components. In addition, a focus of seminoma was found in the lymphoid stroma at the tumour margin (Fig. 2F). These round tumour cells were rich in clear cytoplasm and positive for Oct4, SALL4 (Fig. 2K), PLAP (Fig. 2L) and CD117 and negative for CK, CEA, CD30 and SOX2. No other teratoma components were found except for small focal cartilage.

**Fig. 3:**
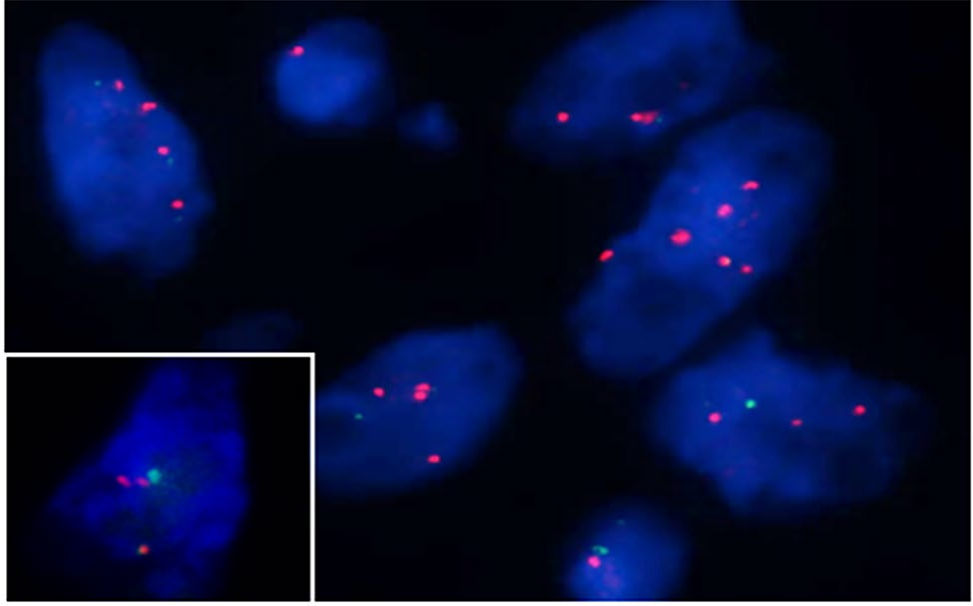
Chromosome 12p copy number increased in VMT cells by FISH (red signals)

KRAS/Control 12 FISH probe group (Empire Genomics) was used to check 12p copy number variation. The DNA probe hybridized with chromosome 12p12.1 and 12p11.1-q11. Three or more KRAS signals observed in a single cell were defined as 12p copy number increase. In our case, 30-40% of VMT cells harbored 12p amplification (Fig. 3), but no positive signals were observed in the seminoma components.

This case was diagnosed as mediastinal high-grade VMT with seminoma. The patient did not undergo preoperative and postoperative chemoradiotherapy and has been disease-free without recurrence and metastasis for 14 months to date. No disease progression was noted by the follow-up serological tests (AFP, HCG, CEA, LDH) or CT scans which were performed three and six months after surgical resection.

## Discussion and Conclusions

The case is the first VMT-seminoma reported. It occurred in a teenage male without any preoperational treatment who had a history of morning glory syndrome.

The concept of VMT was first proposed by Levy in 2021 [3]. Levy et al. reported fifty-five cases of VMT-GCT, and they speculated the VMT and GCT components were closely related because VMT may originate from the splanchnic mesoderm of the embryonic yolk sac, where vasculogenesis initially occurs during embryogenesis. It had been reported that a substantial proportion of mediastinal germ cell tumours with yolk sac tumour elements are neoplastic reiterations of embryonic vasculogenesis [4].

VMT has a distinct angiogenic lineage and is characterized by a spectrum of morphologies that range from the earliest “cleft-formation” stage and the later thick-walled neoplastic vessels, which can be mixed with a neoplastic stroma of varying cellularity. Morphologically, in our case, a similar pattern of angiogenic lineage was also observed, from cleft-like thin-walled to thick-walled neoplastic vessels, which were lined by atypical, nonstratified endothelium in a neoplastic stroma with obvious atypia, but there was no angiosarcoma.

Adequate sampling of the primary mediastinal GCT-VMT is essential because sometimes the exuberant vascular proliferation may be disguised as vascular tumours and cover up GCT components [5], or sometimes postchemotherapy-related changes in mediastinal mixed germ cell tumour masquerade as a vascular neoplasm [6]. In our case, after completely sampling the tumour mass, the seminoma components and a small focal cartilage (possible teratoma) were identified. In cases of small biopsy of mediastinal tumours, due to limited tissue sampling, GCT components may be missed. In this situation, serological information on AFP and β-HCG levels should be helpful for suspicious GCT components.

Chromosome 12p abnormalities mostly present in somatic malignancies of male testicular germ cell tumors [7], and usually present in the form of an isochromosome 12p (i12p) or regional amplification events [8]. Mediastinal GCTs share 12p abnormalities with GCTs at other sites [9, 10]. Levy found an abnormality in 12p in both endothelial and stromal components in 8/9 VMT cases and 1/1 angiosarcoma cases. These findings support VMT is closely related to GCT. In our case, 12p was only identified in VMT cells, but not found in seminoma cells. This may be due to fewer seminoma cells and their scattered distribution in VMT after multiple slices.

Morning glory syndrome (MGS) is a congenital optic disc condition. It is a rare sporadic disorder that was first described in 1970 by Kindler. The pathogenesis of MGS is not fully understood [11]. It was suggested that anomalous mesodermal differentiation can result in MGS [12], or that it is an autosomal dominantly inherited disease caused by a mutation in the MMP19 gene located at chromosome 12q13.2 [13].

VMT is very rare, and there are no specific treatment guidelines for it. Thus, at present, surgical resection is the primary approach, and it can be treated with chemotherapy and/or radiotherapy if necessary, depending on the composition of the GCT and the patient’s condition. However, VMT may be a precursor of angiosarcoma, although this is usually rare. Additionally, VMT may increase the risk of leukaemia or myelodysplastic changes in patients, and regular follow-up is recommended [3]. The patient in our case is still undergoing follow-up examinations and showing no signs of recurrence after the operation without any other treatment.

This VMT-seminoma case may expand the spectrum of GCT-STM. First, GCT components can include seminoma, and second, GCT-VMT may occur in patients without preoperative radiotherapy and/or chemotherapy. Seminoma is sensitive to chemotherapy, which may be the reason why there were only non-seminoma GCT componentsnin Levy’s cohort. We also noticed that one patient who had a preoperational biopsy diagnosis of seminoma in Levy’s cohort.

In conclusion, we reported a new entity of VMT-seminoma broadening the GCT-STM spectrum.

## Data Availability

Not applicable.

## List of Abbreviations

GCT: germ cell tumour
GCT-STM: germ cell tumour with somatic-type solid malignancy
VMT: vasculogenic mesenchymal tumour
AFP: alpha-fetoprotein
β-HCG: beta-human chorionic gonadotropin
CEA: carcinoembryonic antigen
MGS: morning glory syndrome
